# Cyanobacterial Blooms and Microcystins in Southern Vietnam

**DOI:** 10.3390/toxins10110471

**Published:** 2018-11-14

**Authors:** Bui Trung, Thanh-Son Dao, Elisabeth Faassen, Miquel Lürling

**Affiliations:** 1 Aquatic Ecology & Water Quality Management Group, Department of Environmental Sciences, Wageningen University, P.O. Box 47, 6700 AA Wageningen, The Netherlands; Els.Faassen@wur.nl (E.F.); miquel.lurling@wur.nl (M.L.); 2 Institute for Environment and Resources, Vietnam National University—Hochiminh City, Linh Trung Ward, Thu Duc District, Ho Chi Minh City 700000, Vietnam; 3 Hochiminh City University of Technology, Vietnam National University—Hochiminh City, 268 Ly Thuong Kiet Street, District 10, Ho Chi Minh City 700000, Vietnam; dao.son@hcmut.edu.vn; 4 Department of Aquatic Ecology, Netherlands Institute of Ecology (NIOO-KNAW), P.O. Box 50, 6700 AB Wageningen, The Netherlands

**Keywords:** cyanobacteria, cyanotoxins, Mekong river, aquaculture

## Abstract

Studies on cyanobacteria in Vietnam are limited and mainly restricted to large reservoirs. Cyanobacterial blooms in small water bodies may pose a health risk to local people. We sampled 17 water bodies in the vicinity of urban settlements throughout the Mekong basin and in southeast Vietnam. From these, 40 water samples were taken, 24 cyanobacterial strains were isolated and 129 fish, 68 snail, 7 shrimp, 4 clam, and 4 duck samples were analyzed for microcystins (MCs). MCs were detected up to 11,039 µg/L or to 4033 µg/g DW in water samples. MCs were detected in the viscera of the animals. MC-LR and MC-RR were most frequently detected, while MC-dmLR, MC-LW, and MC-LF were first recorded in Vietnam. *Microcystis* was the main potential toxin producer and the most common bloom-forming species. A potential health hazard was found in a duck–fish pond located in the catchment of DauTieng reservoir and in the DongNai river where raw water was collected for DongNai waterwork. The whole viscera of fish and snails must be completely removed during food processing. Cyanobacterial monitoring programs should be established to assess and minimize potential public health risks.

## 1. Introduction

When sufficient resources are available, cyanobacteria may proliferate and bloom in reservoirs, lakes, rivers, estuaries, and coastal systems, where they may cause a multitude of water quality concerns, such as producing malodor, causing nocturnal oxygen deficiency leading to fish kills, disrupting the aquatic ecosystem, and posing a health hazard to wildlife, game, and humans, because of their potential to produce strong toxins (cyanotoxins) [1,2]. Cyanobacteria can cause acute or chronic toxicity to animals and humans via different exposure routes, such as contaminated drinking water, fish or shell fish, through crops irrigated with cyanobacteria-infested waters, or through recreational exposure. Numerous animal poisonings associated with exposure to cyanobacteria have been reported by Hudnell et al., 2008 [3]. The potency of the cyanobacterial toxins is underpinned by the death of 30 kg dogs exposed to anatoxins [4] and microcystins [5]. Three cows and ten calves died in northwest Queensland in 1997 and 148 people were hospitalized in Palm Island in 1979 after expoxure to cyanobacteria [6,7,8]. In total, 458 suspected human illnesses and 175 animal deaths associated with cyanobacterial bloom events have been reported in the U.S.A. during 2007–2011 [9].

Cyanotoxins are produced by several cyanobacterial species amongst others belonging to the genera *Aphanizomenon*, *Anabaena* (*Dolichospermum*), *Anabaenopsis*, *Cylindrospermopsis*, *Microcystis*, *Nostoc*, *Nodularia*, and *Oscillatoria* (*Planktothrix*) [10]. The most widespread and notorious class of cyanotoxins are the microcystins (MCs) that are known as non-ribosomal processed cyclic heptapeptides. The general structure is cyclo(-d-ala^1^-l-X^2^-erythro-β-d-methylaspartic acid^3^-l-Z^4^-Adda^5^-d-isoglutamic acid^6^-*N*-methyldehydroalanine^7^). Adda^5^ is (2S,3S,8S,9S)-3-amino-9-methoxy-2,6,8-trimethyl-10-phenyldeca-4,6-dienoic acid and X and Z are variable l-amino acids on the 2 and 4 positions, which contribute mostly to the dozens of variants of MCs that have been detected. The amino acids on the 3 (d-MeAsp) and 7 (d-Glu6-Mdha) positions can also occur as demethylated variants. MCs are potent inhibitors of protein phosphatases, but the toxicity of different variants to mice varied substantially, where replacement of the hydrophobic leucine (L) in the first variable position with a hydrophilic amino acid (e.g., arginine, R) that dramatically reduces toxicity [11]. 

**Figure 1:**
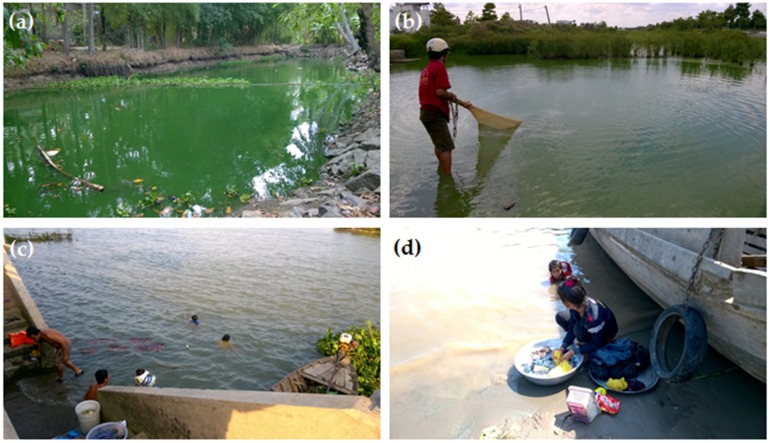
Daily activities of local people potentially exposed to cyanobacterial toxins in the Mekong Delta: Traditional fisheries in cyanobacteria bloom-water ponds (**a**,**b**) and bathing and washing (**c**,**d**).

Southern Vietnam, including the Mekong Delta, is a large area with lakes, ponds, rivers, primary canals, and reservoirs. It includes large systems such as the Mekong river, DongNai river, TriAn reservoir (323 km^2^), DauTieng reservoir (264 km^2^), and BinhThieng reservoir (192 hectare) and numerous smaller canals, streams and fish, shrimp, and duck ponds that are all vulnerable to point source pollution by sewage, ducks, and local fish farming. Consequently, these sites present a high risk for developing cyanobacterial blooms and as they are often in close vicinity to urban settlements, citizens might be at high risk of exposure to cyanobacterial toxins. In aquaculture in the Mekong Delta, cyanobacteria-infested water is commonly treated chemically, i.e., by chlorine or copper sulphate, which may then lead to high water concentrations of dissolved cyanotoxins from cell lysis [12,13]. Hence, fish and other aquatic animals grown in the aquaculture ponds may contain cyanotoxins posing a potential risk to consumers (Figure 1a,b). Surface water collected directly from the water bodies by local water supply stations in the Mekong Delta is generally treated by rock and sand filters in combination with chlorinated disinfection before supplying the water to the local communities [14]. However, when cyanobacterial blooms occur in these water bodies, the presence of cyanotoxins in treated drinking water cannot be totally excluded. Local residents in the Mekong Delta use the surface water for daily bathing and washing (Figure 1c,d). Hence, the presence of cyanobacteria in the water bodies mentioned above might infer a health risk to the local people. However, up-to-date information on cyanobacteria and cyanotoxins in southern Vietnam is very limited.

Studies on cyanobacteria in Vietnam have mainly focused on morphological taxonomy [15,16,17,18,19,20]. Few later studies touched upon microcystins (MCs) producing cyanobacteria in natural lakes and reservoirs and showed the occurrence of microcystin variants MC-RR, MC-dmRR, MC-YR, MC-LA, MC-LY, and MC-WR in isolated cyanobacteria and field samples [21,22,23,24]. A first report on MC accumulation in fish and bivalves was recently published by Pham et al. [25], in which MCs concentrations varying from 0.06 to 3.15 µg MCs/g DW were determined in three fish and two bivalves collected in Dau Tieng Reservoir. The study areas and cyanobacterial samples in those studies are limited to a few water bodies, mainly large reservoirs. However, cyanobacteria blooms in small ponds and canals used for collecting drinking water or to cultivate fish or ducks may also pose a health risk to local people. We hypothesize that the cyanobacteria blooming in these small water bodies produce MCs and that MCs are also present in animals living in these water bodies. The aims of the current study were, therefore, (1) to determine the occurrence of cyanobacterial blooms and MCs; (2) to measure the MC content in isolated cyanobacteria strains; (3) to quantify the MC content in animals living in water bodies suffering from cyanobacteria; and (4) to assess health hazards caused by MC exposure to local people via estimated daily intake (EDI).

## 2. Results 

### 2.1. Physical and Chemical Characteristics and Cyanobacterial Blooms 

The results of the physical and chemical characteristics of 17 water bodies at the moment of our sampling in southern Vietnam are summarized in Table 1. The water temperature was high and ranged from 30 to 37.7 °C, which was due to the sampling that mainly took place during the hot season in Vietnam from January to June in 2015 and 2016. The high temperature was a consequence of the heatwaves recorded in recent years in the Indochina peninsula [26]. Salinity ranged from 0.5‰ to 7.6‰ at the sampling points. High concentrations of TN and TP indicated that the water bodies were eutrophic to hyper-eutrophic [27].

**Figure A1:**
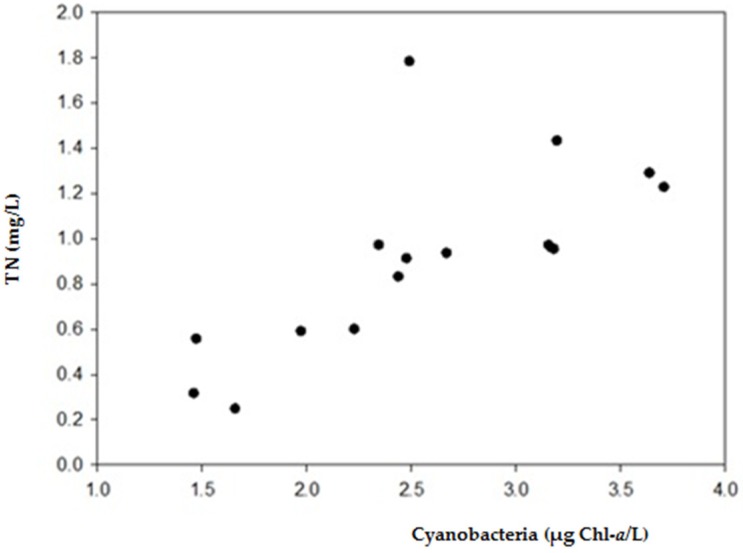
Correlation between cyanobacteria Chl-*a* concentration (µg/L) and TN (mg/L). Pearson = 0.709.

**Figure A2:**
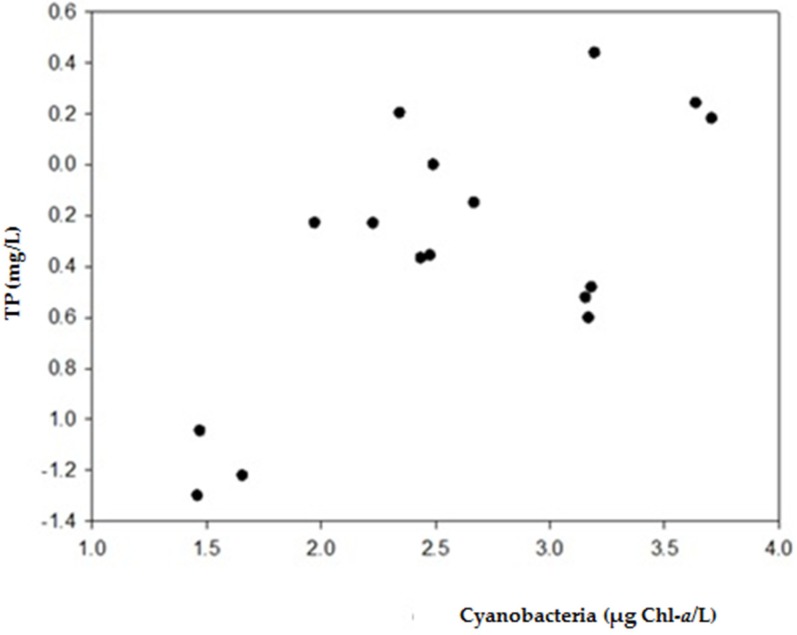
Correlation between cyanobacteria Chl-*a* concentration (µg/L) and TP (mg/L). Pearson = 0.676.

Cyanobacterial blooms were observed in ponds, reservoirs, canals, and rivers in southern Vietnam. The blooms in small ponds, where fish or ducks were cultivated, appeared more dense than the blooms found in reservoirs and rivers that are the main water supplies for domestic purposes, irrigation, and aquaculture. High cyanobacteria chlorophyll-a concentrations from 1437 to 5100 µg chl-*a*/L were measured in several carp ponds in HCMC, in a duck–fish pond and in extensive catfish ponds (Table 1). Lower concentrations from 46 to 420 µg chl-*a*/L were measured in the reservoirs, Tri An, Dau Tieng, and Binh Thieng (Table 1). The abundance of cyanobacteria in terms of chl-*a* was strongly correlated with TN (r = 0.709) and TP (r = 0.676) (Appendix A, Figure A1 and Figure A2). *Microcystis, Planktothrix, Oscillatoria*, and *Cylindropermopsis* were the dominant genera. 

### 2.2. MC Content in Cyanobacterial Field Samples and Isolated Strains

There were 41 field samples collected from the 17 water bodies to determine the MC concentrations. MCs were detected in 28 out of 41 water samples collected in 3 reservoirs, 9 small ponds, 1 canal, 2 lakes, and 2 rivers during bloom events. The MC concentrations of field samples were determined in µg/L and/or µg/g DW and are shown in Table 2. The MC concentrations ranged from under the detection level to 11,039 µg/L or to 4033 µg/g DW. The maximum concentrations of total MC in the water samples were determined in the duck–fish pond in TraVinh province, Mekong delta, where the fish climbing gouramies (*Anabas testudineus*) and ducks were cultivated. A higher MC concentration was detected in 18 samples collected from blooms with *Microcystis* dominance than in the three samples with *Oscillatoria*, *Planktothrix* dominance. Twenty-four cyanobacterial strains were isolated from sampled blooms that were also tested for MC concentration in both µg MC/L and µg MC/g DW and shown in Table A1 and Table A2 (Appendix B). MCs were found in only nine *Microcystis* strains and was not found in four other *Microcystis* strains. In the isolated *Anabaena*, *Anabaenopsis* and *Planktothrix* strains, no MCs were detected (Appendix B). 

MC-LR and MC-RR variants were most frequently detected in 24 and 20 out of 28 MC-containing field samples, respectively. They were also the most abundant MC variants in the samples, in which MC-RR contributed from 44% to 100% of the total MC-pool in 17 samples and MC-LR contributed from 43% to 100% of the total MC-pool in the remaining 11 samples. Our study contributed to the diversity of MC variants as three MC variants including MC-dmLR, MC-LW, and MC-LF were recorded for the first time in Vietnam (Table 3).

### 2.3. MCs Accumulation in Animals

The MC content was determined in 212 organ and tissue samples of aquatic animals, including 129 fish, 68 snail, 7 shrimp, 4 clam, and 4 duck samples collected in water bodies suffering from cyanobacterial blooms. MCs were detected in 36 samples (17% of total samples), including 23 snail, 12 fish, and 1 shrimp samples (Table 4, Appendix C). 

The MC content in the animals varied among fish species, organs, tissues, and seemed to be influenced by the sampling sites where they had been collected (Table 4). On average, the MC content was higher in fish than in shrimp and snail with 24.1, 15.2, and 1.1 µg/g DW in fish, shrimp, and snail, respectively (Table 4). MCs were mainly found in the viscera including the visceral mass, liver, and gut in apple snails, tilapias, suckermouth catfish, goldfish, common carp, ganges river sprat fish, snakeskin gourami fish, and white leg shrimp. MCs were also found in 4 flesh samples, in 3 apple snails, and 1 suckermouth catfish (Table 4). No MCs were found in clam and duck. The highest MC content of 115.95 µg/g DW was detected in the gut of the omnivorous tilapia collected in a fish pond in BinhChanh, which was followed by 108.28 µg/g DW found in the gut of suckermouth catfish collected in the same pond. A lower MC content was found in the gut of goldfish (18.57 µg/g DW) collected in BinhChanh and in the gut of tilapia collected in TriAn, DauTieng reservoirs (11.55 and 0.76 µg/g DW). A trace amount of 0.36 µg MC/g DW was found in the gut of common carp (*Carassius* sp.) collected in BinhChanh. 

MCs were also detected in the livers of tilapia (13.39 µg/g DW) and suckermouth catfish (18.97 µg/g DW) collected in BinhChanh. In our survey, the visceral mass in small tilapia, snakeskin gourami fish, ganges river sprat fish, white leg shrimps, and apple snails was collected to determine the MC content, because it was very hard to separate gut or liver organs from the rest visceral organs of these small animals. In addition, the animals were eaten whole by local consumers, thus the MC content in the visceral mass would be more reliable for warning the local consumers. The MC content in the visceral mass in snakeskin gourami fish and white leg shrimp was 13. 86 and 15.21 µg/g DW; those in tilapia, ganges river sprat fish, and apple snail were 2.87, 2.38, and 0.21–2.90 µg/g DW, respectively. 

MC-LW and MC-LF seem to be more toxic than MC-LR that is currently used for risk calculations and assessments [39,40]. These MC variants were found for the first time in Vietnam, in TriAn reservoir and Mekong, DongNai rivers. Since different MC-variants have different toxicity as determined by bioassays [11,39,40], the contribution of each variant to the overall MC toxicity of the samples was estimated by multiplying its concentration by a toxicity factor as mentioned in Table 1 in Faassen and Lürling 2013 [41]. As a consequence of the lower toxicity factor of MC-RR in comparison with MC-LR (see Table 1 in Faassen and Lürling 2013 [41]), the overall toxicity of MC-RR-dominated samples became less. For instance, the MC concentration in the sample collected in a duck–fish pond in Travinh was 11,039 µg/L, but the overall estimated toxicity of the sample was equal to only 963 µg/L MC-LR equivalents. Samples containing MC-LW and MC-LF were estimated to increase in toxicity when expressed as MC-LR equivalents, because these two variants have higher toxicity factor in comparison with MC-LR. 

**Figure 2:**
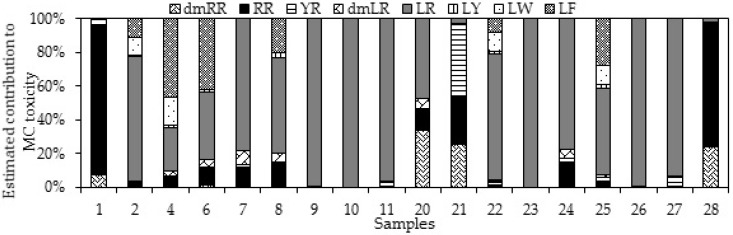
Estimated contribution of eight microcystin (MC)-variants to the total MC toxicity in 18 surface water samples with cyanobacterial bloom in southern Vietnam.

MC-LR occupied, on average, 81% of total toxicity in 13 out of 18 samples containing high MC concentrations (Figure 2). MC-RR and MC-LF contributed 81% and 44%, respectively, to the total toxicity in two other samples. 

## 3. Discussions

### 3.1. Occurrence of Cyanobacterial Blooms

There are few reports on cyanobacterial blooms and MCs in southern Vietnam, such as in TriAn and DauTieng reservoirs [22,25,30,42,43]. In addition to these relatively large water bodies, our study also points out that MC-producing cyanobacterial blooms occur in small ponds, small reservoirs and rivers in southern Vietnam. In fact, this is the first report on cyanobacterial blooms and MCs in the Vietnamese Mekong delta (Table 3). There are thousands of small ponds or water bodies in southern Vietnam, which are cultivating fish and ducks in a combination model to provide daily food for local markets. Pellet feeds containing high levels of nitrogen and phosphorus are used as food for fish and ducks leading to high concentrations of nitrogen and phosphorus from uneaten pellet feeds and feces of fish and ducks. Additionally, chicken manure is often applied to enhance primary productivity during the initial phase of fish cultivation. Our monitoring indicated that the ponds were highly eutrophic or hyper-eutrophic, as TN and TP ranged from 3.98 to 60.7 mg/L and from 0.25 to 1.75 mg/L, respectively (Table 1). The excessive nutrients, high water temperature, and rather stable water column resulted in cyanobacterial blooms in the ponds. These blooms were evidently linked to TN and TP concentrations as the higher the TN and TP concentrations, the higher the cyanobacteria biomass in water (Appendix A). *Microcystis* strains isolated from the ponds expressed better growth rates under warming temperature [44]. Hence, excessive nutrients and high temperature may support the cyanobacterial blooms observed during the dry, hot seasons in 2015 and 2016 in southern Vietnam.

### 3.2. MC Content in Cyanobacterial Field Samples and Isolated Strains

Our study confirmed that *Microcystis* was the main potential toxin producer and the most common bloom-forming species in southern Vietnam, which is in accordance with previous studies [21,22,24,28,29,30,41,43]. The other bloom-forming species, such as *Oscillatoria* and *Planktothrix*, can also accumulate high biomass in the water bodies, but the MC concentrations were below the detection level or found at trace levels (<0.5 µg/L). This finding is supported by the study of Nguyen et al. [28] in several water bodies in Hue, middle Vietnam, where the MC concentrations were high (47.8 µg/L) in bloom samples with *Microcystis* dominance, while the MC concentrations were much lower ranging from below the detection level to 0.05 µg/L in bloom samples with *Arthrospira*, *Merismopedia* dominance, or 1.31 µg/L in bloom samples with *Jaaginema*, *Oscillatoria* dominance [28]. 

### 3.3. MC Accumulation In Animals

**Figure 3:**
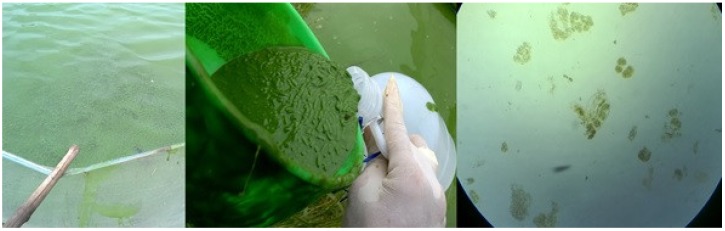
Microscopy revealed that fecal pellets of fish in a fish pond in BinhChanh contained undigested *Microcystis* and rotifer.

In general, the MC content detected in the gut, liver and visceral mass of fish in our study was high when compared to other studies (Table 4). For instance, the MC content in the visceral mass of golden carp collected in BinhChanh was nine times higher than that found in the intestine of golden carp collected in lake Taihu, China [32]. The MC content in the liver of tilapia collected in BinhChanh was almost double that measured in the liver of tilapia collected in Funil and Furnas reservoirs, Brazil [35] and 57 and 87 times higher than the MC content found in the liver of tilapia collected in lake Mburo and lake Victoria, Uganda [36]. Both carp and tilapia are omnivorous fish; when living in ponds with a high biomass of toxic *Microcystis*, they likely consume *Microcystis* via their daily food and consequently may accumulate MCs. Fecal pellets of the fish in BinhChanh fish pond were collected during our sampling and microscopy revealed that the fecal pellets contained undigested *Microcystis* and empty rotifer (Figure 3). Although we cannot estimate how much *Microcystis* was ingested and digested by the fish, the microscopy indicated that *Microcystis* was ingested by the fish. 

Nevertheless, the MCs were under the level of detection in muscle tissues in most of the samples except for suckermouth catfish and snails. The MCs in the muscle tissue of tilapia collected in DauTieng reservoir were under the level of detection (Table 4), which was also found by Pham et al. [25]. However, covalently bound MCs were found in the muscle tissue of tilapia collected in DauTieng reservoir [25]. The hot methanol extraction applied in our study was not able to detect the bound MCs, which may be one of the reasons why MCs were undetected in muscle tissue in the fish in our study.

### 3.4. Public Health Risk Assessment

#### 3.4.1. Risk Assessment for Drinking Water Supplies

DongNai, SaiGon rivers and DauTieng reservoir are the main water supplies for HoChiMinh city (hereafter HCMC) [45,46]—a megacity with 10 million inhabitants. Our monitoring indicated that MC concentrations ranged from 0.09 to 2.22 µg/L—equivalent to 0.01–0.78 µg MC-LR equivalent/L in raw water collected at five sampling points in DauTieng reservoir, which is below the WHO guideline value of 1.0 µg MC-LR/L for drinking water [1]. Additionally, extracellular MCs in raw water in DauTieng reservoir were lower than the level of detection. DauTieng reservoir was a safe source of drinking water at the time of our monitoring, in accordance with WHO guidelines. However, the occurrence of MCs in this reservoir should be paid attention due to their potential health risks. In addition, concentrations of 30 µg/L or 8.55 µg MC-LR equivalent/L and 485 µg/g DW or 47.73 MC-LR equivalent/g DW were detected in samples of a duck–fish pond located in the catchment of DauTieng reservoir. These concentrations were much higher than the WHO guideline value for drinking water. As these duck and fish cultivations occurred in the catchment of DauTieng reservoir, discharged water from these ponds could not only inoculate or increase blooms of cyanobacteria, but also imply a potential health hazard for drinking water supply. 

**Figure 4:**
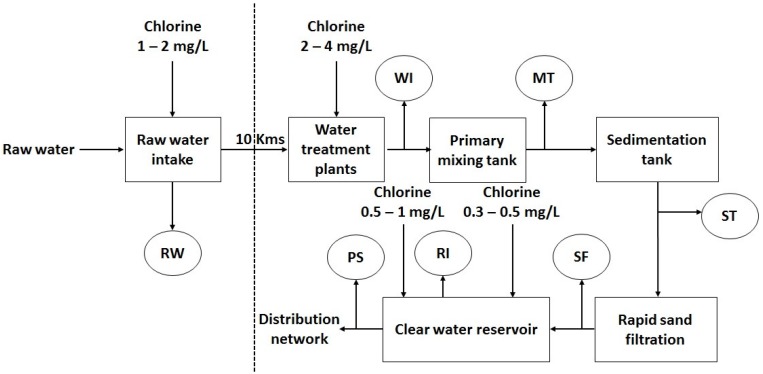
The typical process scheme of a water drinking plant in Vietnam. Modified from Nha Trang et al., 2012 [47]. RW: Raw water; WI: Effluent of pre-chlorination; MT: Effluent of mixing tank; ST: Effluent of the sedimentation tank; SF: Effluent of rapid sand filter; RI: The inlet of the clean water reservoir; PS: The outlet of the clean water reservoir.

An MC concentration of 664 µg/g DW was detected at HoaAn in the Dongnai river, where raw water was collected for DongNai waterwork. This raw water is treated two times with chlorine 1–2 mg/L and 2–4 mg/L to kill algae and moss as in the typical process scheme of a water drinking plant in Vietnam (Figure 4) [47]. Chlorine is known as an algaecide and chlorination has a strong action on membrane disruption, which can induce cyanobacterial cell lysis. For instance, 97% of *M. aeruginosa* cells was lysed within the first minute when exposed to 3 mg/L chlorine [12]. Chlorine application of 2–4 mg/L during pre-chlorination in many drinking water plants in Vietnam can result in cell lysis and consequently cyanotoxins can be liberated into drinking water. Although there was no information on cyanobacteria concentration in bloom events in HoaAn in January 2016, the greenish appearance and *Microcystis* dominance in water with an MC content of 664 µg/g DW or 554 µg MC-LR equivalent/g DW should be considered as a potential health hazard for water consumers.

Rivers, lakes, and ponds in the Mekong delta are among the main water supplies for household daily use. These water supplies provided 36% for households’ daily demands (besides drinking and cooking) and 25% for the demands of drinking and cooking [48,49] for 17.4 million people. The available surface water from these water supplies can be directly used for domestic purposes, especially in the dry season and cyanobacteria accumulation in the surface water has not been recognized as a potential health risk to local people who settle in villages and cities along and above the rivers, lakes and ponds. Additionally, raw water for local water supply stations in the Mekong delta collected from the rivers and canals in this area is treated via a simple process—rock and sand filters in combination with chlorinated disinfection—prior to reaching the local communities [14]. The piped-water provided by these local water supply stations may be contaminated by MCs liberated from cyanobacterial cell lysis due to the chlorination in the water process. Therefore, additional studies should examine the processed drinking water in the presence of MCs.

#### 3.4.2. Risk Assessment for MC-Contaminated Foods

We assessed public health risk for the MC-contaminated apple snails and fish by calculating the estimated daily intake (EDT) for digestion of apple snails and fish (Table 5). The estimated daily intake (EDI) of MC-LR equivalents (µg/kg/day) is based on the tolerable daily intake (TDI) of 0.04 µg MC-LR per kg of body weight per day over the lifetime of an individual weighing 60 kg and eating 300 g snail or 100 g fish per day, as recommended by the WHO [1]. DW of fish in our study was converted to WW by a conversation factor of 0.311 [34,37,38]. WW of apple snails was estimated by DW and 83% water content in live apple snails [50].

The golden apple snails, *Pomacea canaliculata* and *Pomacea maculata*, are invasive freshwater snails in Vietnam. The snails were introduced for culture as a food source in 1988, but the snails then spread in rice fields, canals, and rivers, becoming a harmful pest in wetland rice culture and other crops [51,52]. The apple snails are rich protein and mineral sources, hence, they are promoted for use as food for humans and as live-food in aquaculture and agriculture. For example, the apple snails are applied as food for striped catfish (*Pangasianodon hypophthalmus*) fingerlings [53] or as a protein supplement to replace soya bean meal in the diets of ducks in the Mekong delta [54]. The apple snails are being sold on many markets throughout Vietnam and have become a common food for the Vietnamese. Our study indicated that apple snails collected from water bodies with a cyanobacterial bloom contained MC both in their visceral and muscle tissue. The calculated EDI indicated that the viscera of apple snails had accumulated MCs (as MC-LR equivalent) 3 to 50 times higher than the TDI value recommended by the WHO. Therefore, it is important to test the viscera of apple snails collected in water bodies with toxic cyanobacteria on accumulated MCs, since evidently apple snails may pose a health hazard for human consumption in southern Vietnam. MC-LR equivalents found in the muscle tissues of apple snails collected in TriAn reservoir were almost equal to the TDI value. Hence, the consumption of apple snails in southern Vietnam is safer for humans if their viscera is completely removed and no more than 300 g flesh of the snails is consumed.

Ganges river sprat, sknakskin gourami fish, and tilapia are common food for local people in southern Vietnam. Ganges river sprat and sknakskin gourami fish are small-sized, high-value fish and well known as delicious specialties in the Mekong Delta and TriAn reservoir. The whole body of fresh and dried Ganges river sprat and sknakskin gourami fish are eaten by consumers, because time-consuming and labour-intensive actions are required to remove the viscera of these small fish. Moreover, the lipids accumulated in the viscera of these fish also contribute to its delicious flavour. However, the EDI calculated from MC-LR equivalents found in the viscera of Ganges river sprat and sknakskin gourami fish were 16 and 179 times higher than the recommended TDI value, respectively. The EDI values of the visceral mass in tilapia collected in DauTieng and TriAn reservoir were also 3 and 10 times higher than the recommended TDI value, respectively. The EDI value of the gut in tilapia collected in BinhChanh fish pond was 1440 times higher than the recommended TDI value.

The viscera of the fish can account for 10–18% of the whole body weight [55]. Although MCs were not detected in the flesh, the high MC concentrations in the viscera of the fish could possibly pose a health risk to consumers eating the whole fish. It is highly recommended that the viscera and especially, the gut of ganges river sprat, sknakskin gourami fish, and tilapia are removed before the fish is consumed.

The suckermouth catfish is an ornamental fish, but it is also known as an invasive species in fresh and brackish water systems in Vietnam. The invasion of suckermouth catfish in the Mekong Delta has been reported by public media since 2004 [56]. The suckermouth catfish has been sold on local markets as food since 2011 [57]. In particular, the suckermouth catfish was recently used as food for patients with diabetes due to local people believing that the flesh of the fish can reduce diabetic signs; this resulted in more serious diabetes, as reported by Faculty of Endocrinology at Hospital of 115 in HCMC [58]. The MC concentration found in the gut of suckermouth catfish was very high, leading to an EDI that was 1305 times higher than the recommended TDI value. Nevertheless, the gut of suckermouth catfish is completely removed during processing and thus no health hazard is expected from ingesting it. However, the flesh and liver of the catfish showed an EDI 48 and 236 times higher than the recommended TDI value and thus it could be considered a high potential hazardous food.

#### 3.4.3. Cyanobacteria Control in Southern Vietnam

There is currently no management strategy to control cyanobacteria in southern Vietnam. This is due to the fact that cyanobacterial blooms have been considered as harmless phenomena. No information on the acute and long-term impacts of MCs on the health of the local public has been reported in this area. However, this and other studies [22,25,30] show that MCs were found in water and in aquatic animals with concentrations and contents that could be assessed as potentially hazardous to the health of the local public. Therefore, we suggest that cyanobacteria-monitoring programs should be established to identify the spatial and temporal variability of cyanobacterial contamination, to assess the potential hazards to the health of the local public and to warn local people who are at significant potential risk of exposure to cyanobacterial toxins. With predicted climatic changes in southern Vietnam, this becomes even more important, especially as warming temperature can boost the high accumulation of cyanobacterial biomass in water bodies in southern Vietnam.

## 4. Conclusions

Eutrophication and hyper-eutrophication with the accumulation of high cyanobacterial biomass were observed in several water bodies (reservoirs, rivers and small ponds) in southern Vietnam. Eutrophication and cyanobacterial blooms in small ponds, where fish and/or ducks were cultivated, were more serious than those in reservoirs and rivers. *Microcystis* was the main potential toxin producer and the most common bloom-forming species in southern Vietnam. The MC concentrations ranged from <LOD to 11,039 µg/L or to 4033 µg/g DW in field samples. MCs were only found in isolated *Microcystis* strains and were <LOD in other isolates, including *Anabaena*, *Anabaenopsis*, and *Planktothrix* strains

MC-LR and MC-RR variants were most frequently found and the most abundant MC variants in MC-containing field samples. Three MC variants—MC-dmLR, MC-LW, and MC-LF—were recorded in Vietnam for the first time. MC-LR, MC-RR, and MC-LF significantly contributed to the total toxicity of MC-containing samples.

The MC content in fish was higher than in shrimp and snail. MC was mainly found in the visceral mass, liver, and gut, so consuming whole MC-containing fish and snails is not safe. It is strongly recommended that the whole viscera of fish and snails must be completely removed during food processing, especially when the animals are collected from water bodies with a high cyanobacteria biomass. The suckermouth catfish should be considered as an ornamental fish, while it is not a safe food source.

Cyanobacterial monitoring programs should be established to assess and minimize potential public health risks.

## 5. Materials and Methods

### 5.1. Sampling

**Figure 5:**
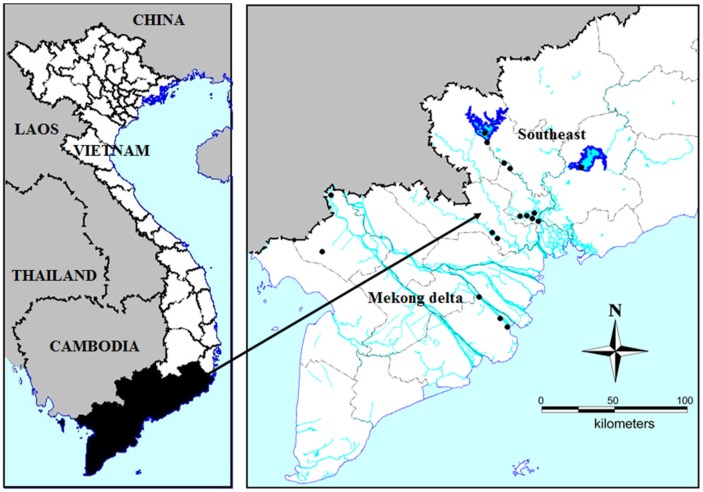
Locations of the sampling sites in South Vietnam. Black dots indicate positions where cyanobacterial blooms occur and the samples were collected.

Seventeen water bodies including rivers, lakes, ponds, canals, and reservoirs in the vicinity of urban settlements throughout the Mekong basin, and in Southeast Vietnam (Figure 5) were sampled once during the dry season (December–May/June) to assess blooms, potential eutrophication effects and resulting cyanobacteria and MCs.

At each collecting site, temperature, salinity, and pH were measured by pH/Cond 340i meter (WTW, Weilheim, Germany). Cyanobacterial-chlorophyll-a was measured with the bbe AlgaeTorch which is a lightweight instrument for the simultaneous quantification of the chlorophyll-a content of cyanobacteria and the total chlorophyll content of microalgae in water. (bbe Moldaenke GmbH, Schwentinental, Germany). Samples from sampling sites where Chl-*a* was higher than 200 µg/L were measured in a bucket after the dilution of collected scum material with tap water to maintain the advised measuring range for the AlgaeTorch. Cyanobacterial scum samples were also collected for isolation and filtration; sub-samples were preserved (in Lugol’s iodine) for microscopic analysis (dominant cyanobacterial species). In addition, 1 to 300 mL of surface water from water-bloom sites was filtered through GF/C filters. The filters and filtrates were stored at −20 °C upon MC analysis. Animals in infested water bodies with cyanobacterial bloom were collected to determine MCs in their tissues.

Samples for nutrient analysis were kept on ice and transported within 24 h to the laboratory of Water Quality, Institute for Environment and Resources where nutrients were analyzed colorimetrically with a spectrophotometer (DR/2010, Hach, Loveland, CO, USA) using the following APHA (2005) [59] methods: Nitrate 4500NO_3_^−^, ammonium 4500NH_4_^+^, total nitrogen (TN) Kjeldahl 4500N, phosphate, and total phosphorus (TP) 4500P. The detection limits of the equipment for these parameters were 0.02 mg/L (nitrate), 0.04 mg/L (ammonium), 0.06 mg/L (TN Kjeldahl), and 0.05 mg/L for both TP and phosphate.

### 5.2. Strains Isolation

In the laboratory, single *Microcystis*, *Planktothrix*, *Anabaenopsis*, *Anabeana* cells or colonies were picked out of the collected scum material by the micropipette-washing method [60]. These isolates were grown in small glass tubes with a few mL modified WC medium (Woods Hole modified CHU10-medium) [61] for several months at 25 °C, under a 14:10 h light/dark cycle at a light intensity of 70 µmol photon/m/s. When isolates reached a greenish appearance, they were transferred into 50 mL Erlenmeyer flasks and subsequently into 250 mL flasks. In total, there were 24 isolated strains (Table A1 and Table A2, Appendix B).

### 5.3. MC Analysis

The frozen filters stored at −20 °C were transferred to 8 mL glass tubes and dried for two hours in a freeze-drier (Alpha 1-2 LD, Martin Christ Gefriertrocknungsanlagen GmbH, Osterode am Harz, Germany). Tissue and scum samples were also dried for several hours in the freeze-drier and 5 to 8 mg freeze-dried material was then transferred to 2 mL Eppendorf vials.

The filters, the tissue and scum samples were extracted three times at 60 °C in 2.5 and 0.5 mL 75% methanol and 25% Millipore water (*v*/*v*). The extracts were then dried in the Speedvac (Savant SPD121P, Thermo Scientific, Waltham, MA, USA) and subsequently reconstituted in 900 μL 100% methanol. The reconstituted samples were transferred to 2 mL Eppendorf vials with a cellulose-acetate filter (0.2 μm, Grace Davison Discovery Sciences, Deerfield, IL, USA) and centrifuged for 5 min at 16,000× *g* (VWR Galaxy 16DH, VWR International, Buffalo Grove, IL, USA). Filtrates were then transferred to amber glass vials for LC-MS/MS analysis. If needed, samples with high MC concentrations were diluted in methanol before re-analysis.

Concentrations of eight MC variants (dm-7-MC-RR, MC-RR, MC-YR, dm-7-MC-LR, MC-LR, MC-LY, MC-LW, and MC-LF) and nodularin (NOD) were determined by LC-MS/MS as described in [5,62]. LC-MS/MS analysis was performed on an Agilent 1200 LC and an Agilent 6410A QQQ (Agilent Technologies, Santa Clara, CA, USA). The MCs were separated on an Agilent Eclipse 4.6 × 150 mm, 5-µm column. Hereto, a 10 µL sample was injected; the flow rate was 0.5 mL/min; the column temperature was 40 °C. Eluents were Millipore water with 0.1% formic acid (*v*/*v*, Eluent A) and acetonitrile with 0.1% formic acid (*v*/*v*, Eluent B) that were run using an elution program of 0–2 min 30% B, 6–12 min 90% B, with a linear increase of B between 2 and 6 min and a 5-min post run at 30% B. Detailed information on MS/MS settings for each MC can be found in [41]; information on the recovery, repeatability, limit of detection, and limit of quantification of the analysis is given in [5]. MCs were quantified against certified standards that were obtained from DHI LAB Products (Hørsholm, Denmark).

## Figures and Tables

**Table 1:** Water temperature (°C), salinity (‰), pH, total nitrogen (TN), total phosphorus (TP), ammonium-N, nitrate-N, and phosphate-P concentrations in 17 sampling sites as shown in Figure 5. Also indicated are the chlorophyll-a concentrations (Chl-*a*, µg/L) and the dominant cyanobacteria genera (Domi.), where Mic = *Microcystis*, Plank = *Planktothrix*, Cyl = *Cylindrospermopsis*, and Osc = *Oscillatoria.*

Location	Water Body	Domi.	Temp. (°C)	Sal. (‰)	pH	TN (mg/L)	TP (mg/L)	N-NH_4_ (mg/L)	N-NO_3_ (mg/L)	P-PO_4_ (mg/L)	Chl-*a* (µg/L)
TraVinh	Duck–fish pond	Mic	33.1	0.5	9.58	19.5	1.75	1.14	0.28	0.02	4352
TraVinh	Mekong river	Mic	33.2	6.7	7.88	2.07	0.05	BLD	0.08	0.02	28.9
TraVinh	Shrimp pond	Plan	27.5	1.0	8.75	8.19	0.44	0.83	0.08	BLD	300
LongAn	Wastewater pond	Plan	31.5	0.0	9.22	27.1	2.75	2.12	0.31	0.43	1572
LongAn	Duck pond	Plan	31.2	0.0	9.17	60.7	1.00	1.06	0.14	0.06	310
BinhChanh	Fish pond	Mic	37.5	7.4	9.89	9.13	0.25	0.57	BLD	BLD	1480
BinhChanh	Fish pond	Mic	37.4	7.5	9.77	9.00	0.33	0.67	BLD	BLD	1520
BinhChanh	Fish pond	Mic	37.4	7.4	9.79	9.36	0.30	0.46	BLD	BLD	1437
BinhChanh	Fish pond	Mic	37.7	7.6	9.66	16.91	1.52	1.02	0.04	0.19	5100
BinhChanh	*Wastewater* canal	Mic	36.0	7.0	9.00	8.62	0.71	0.55	BLD	BLD	467
CuChi	Rice farm	Cyl	34.9	0.0	9.13	6.78	0.43	0.53	0.23	BLD	274
CuChi	Fishing pond	Plan	34.3	0.0	7.91	3.6	0.09	0.17	0.12	BLD	29.7
An Giang	reservoir	Osc/Plan	33.6	0.0	7.96	1.77	0.06	0.96	0.18	0.03	45.6
An Giang	Catfish pond	Plan	30.0	0.1	7.31	9.38	1.60	3.96	0.1	1.04	221
DauTieng	Duck–fish pond	Mic	33.5	0.0	9.82	3.98	0.59	0.39	0.14	0.10	169
DauTieng	reservoir	Mic	34.0	0.0	8.12	3.89	0.59	0.39	0.14	0.10	94
TriAn	reservoir	Mic	34.2	0.0	8.57	15.5	0.84	-	0.4	0.10	420

**Table 2:** Total microcystin (MC) concentrations (µg/L and/or µg/g DW) of samples collected at sampling sites where cyanobacteria blooms were found, including the chlorophyll-a concentrations (Chl-*a*, µg/L) and the dominant cyanobacteria genera (Domi.), where Mic = *Microcystis*, Plank = *Planktothrix*, and Osc = *Oscillatoria.* a-indicates not determined, <LOD = below level of detection.

Location	Water Body	Domi. Genera	Chl-*a*	MC Concentration	
			µg/L	µg/L	µg/g DW
TraVinh	Duck–fish pond	*Mic*	4352	11,039	4033
LongAn	duck pond	*Osc*	310	0.18	<LOD
Mekong-TraVinh	river	*Mic*	29	57	2591
TriAn-DongNai	reservoir	*Mic*	187	2610	-
DauTieng-TayNinh	reservoir	*Mic*	169	30	-
DauTieng-TayNinh	reservoir	*Mic*	94	-	485
BinhChanh	Fish pond	*Mic*	5100	821	1156
BinhChanh	Fish pond	*Mic*	1520	293	1477
CuChi	Canal	*Plank*	8	0.03	-
CuChi	Fish pond	*Plank*	30	0.08	-
An Giang	Reservoir	*Plank*	46	0.31	-
XuanHuong-DaLat	Lake	*Mic*	-	-	6
TuyenLam-DaLat	Lake	*Mic*	-	-	278
DongNai	River	*Mic*	-	-	602–664

Mic: *Microcystis* sp., Osc: *Oscillatoria* sp., Plank: *Planktothrix* sp.

**Table 3:** MC variants and concentration in water bodies in Vietnam.

Location Water Bodies	Species/Genera Dominant	MC Variants	Dominant Variants	Microcystin Concentration		Ref.
				µg/L	µg/g DW	
Lake Thanh Cong, Ha Noi	*M. aeruginosa*	MC-RR, MC-YR, MC-WR, MC-dmRR, MC-dmWR, MC-LR	MC-WR		4240	[21]
Pond and rivers in Hue	*M*. spp.	MC-RR, MC-LR	MC-RR, MC-LR	1.02–76.20		[28]
TriAn reservoir	*M. botrys* *M. wesenbergii*	MC-LR, MC-RR, MC-LA, MC-LY,	MC-RR MC-LR		450–640	[22]
NuiCoc resrvoir	*M. aeruginosa,* *M. wesenbergii,* *M. botrys*.	MC-RR MC-LR	MC-RR MC-LR		45.4–1699	[24,29]
DauTieng reservoir	*Microcystis aeruginosa*	MC-LR, MC-RR, MC-YR	MC-RR		521–669	[30]
Duck–fish pond TraVinh	*Microcystis* spp.	MC-dmRR, MC-RR, YR and dmLR	MC-RR	7771–91,721	407–4033	This study
Mekong river TraVinh	*Microcystis* spp.	MC-dmRR, MC-RR, YR and dmLR, MC-LR, MC-LY, MC-LW, MC-LF	MC-LR	57.26	1575–2591	This study
TriAn reservoir	*Microcystis* spp.	MC-RR, dmLR, MC-LR, MC-YR, MC-LY, MC-LW,	MC-LR	425–3619		This study
DauTieng reservoir	*Microcystis* spp.	MC-dmRR, MC-RR, dmLR, MC-LR	MC-RR	0.09–30	485	This study
Fish pond BinhChanh	*Microcystis* spp.	dmLR, MC-LR, MC-YR		293–821	1156–1477	This study
XuanHuong Lake	*Microcystis* spp.	MC-LR	MC-LR		6	This study
TuyenLam Lake	*Microcystis* spp.	MC-RR, MC-YR, dmLR, MC-LR	MC-RR		278	This study
DongNai river	*Microcystis* spp.	MC-dmRR, MC-RR, MC-YR, dmLR, MC-LR, MC-LY, MC-LW, MC-LF	MC-LR		602–664	This study

**Table 4:** MC concentrations accumulated in various organs in aquatic animals.

Organism/Species	Organ	MC Concentration (µg/g DW)	Location	Ref.
Fish				
Common carp *Cyprinus carpio*	G, M	0.36; UD	BinhChanh	This study
	M	0.060	Fishponds, Serbia	[31]
	M, L, K, I, H	0.0317; 0.0295; 0.0323; 0.133; 0.019	Lake Taihu, China	[32]
	M, L	0.69–3.45; 1.09–2.05	Eğirdir, Turkey	[33]
Golden carp *Carassius auratus*	VM, M	18.57, UD	BinhChanh	This study
	M, L, K, I, H	0.0267, 0.0454, 0.114, 2.04, 0.0595	Taihu, China	[32]
Suckermouth catfish (*Hypostomus punctatus*)	G, L, M	108.38; 18.97; 3.73	BinhChanh	This study
Tilapia (*Oreochromis* sp.)	G; L, M	115.95; 13.39; UD	BinhChanh	This study
	G, VM, L, M	11.55; 2.87; UD; UD	TriAn reservoir	This study
	G, M	0.76; UD	DauTieng reservoir	This study
	L, I, M	2.1; 2.6; UD	DauTieng reservoir	[25]
	L, M	0.0562; 0.0134–0.0168	Fish pond, Southeast Asian	[34]
	L	6.752 *–8.682 *	Funil and Furnas Reservoirs, Brazil	[35]
	G, L, M	4.756 *; 0.154 *; 0.029 *	Lake Victoria, Uganda	[36]
	G, L, M	4.219 *; 0.235 *; 0.672 *	Lake Mburo, Uganda	[36]
Snakeskin gourami (*Trichogaster pectoralis*)	VM, M	13.86; UD	BinhChanh	This study
Ganges river sprat (*Corica soborna*)	VM, M	2.38, UD	TriAn reservoir	This study
**Snails**				
Apple snail (*Pomacea canaliculata*)	VM, M	0.21–3.21; 0.15–0.30	TriAn reservoir	This study
	VM	1.61–2.90	BinhChanh	This study
**Shrimp**				
White leg shrimp (*Litopenaeus vannamei*)	VM, M	15.21; UD	TraVinh	This study

G = gut; L = Liver; VM = visceral mass; M = muscle; H = heart; I = intestine; K = kidney; UD = undetectable; conversted to DW by WW * a conversation factor of 0.311 [34,37,38].

**Table 5:** Estimated daily intake (EDI) of MC-LR equivalents (µg/kg/day) in snails and fish collected at different locations in southern Vietnam.

EDI	Muscle Tissue	Visceral Mass	Gut	Liver	Egg	Head
Snails-TriAn	0.039	0.107	-	-	0.038	-
Snails BinhChanh	0	1.997	-	-	0	-
Ganges river sprat-TriAn	0	0.618	-	-	-	-
Sknakskin gourami fish-BinhChanh	0	7.155	-	-	-	0
Tilapia-TriAn	0	0.119	1.111	-	-	-
Tilapia-DauTieng	0	0.393	-	-	-	-
Tilapia-BinhChanh	0	-	57.61	6.819	-	-
Suckermouth catfish-BinhChanh	1.903	-	52.19	9.441	-	-
TDI recommended by WHO	0.04	0.04	0.04	0.04	0.04	0.04

## Appendix B

**Table A1:** Microcystin concentration (µg/L) in isolated cyanobacteria.

No.	Name	dmRR	RR	NOD	YR	dmLR	LR	LY	LW	LF	Total
1	*Microcystis* sp.1	UDL	UDL	UDL	0	19.62	2255.31	UDL	UDL	UDL	2274.94
2	*Microcystis* sp.2	UDL	UDL	UDL	5.87	6.55	67.08	UDL	UDL	UDL	79.50
3	*Microcystis* sp.3	UDL	UDL	UDL	0	6.12	1693.98	UDL	UDL	UDL	1700.10
4	*Microcystis* sp.4	UDL	UDL	UDL	539.80	4.93	336.06	UDL	UDL	UDL	880.79
5	*Microcystis* sp.5	UDL	UDL	UDL	UDL	0.66	1368.22	UDL	UDL	UDL	1368.88
6	*Microcystis* sp.6	UDL	UDL	UDL	UDL	UDL	UDL	UDL	UDL	UDL	UDL
7	*Microcystis* sp.7	UDL	UDL	UDL	UDL	UDL	1.22	UDL	UDL	UDL	1.22
8	*Microcystis* sp.8	UDL	UDL	UDL	UDL	UDL	UDL	UDL	UDL	UDL	UDL
9	*Microcystis* sp.9	5.10	19.03	0.03	0.21	1.32	9.67	UDL	UDL	UDL	35.36
10	*Microcystis* sp.10	UDL	UDL	UDL	UDL	UDL	UDL	UDL	UDL	UDL	UDL
11	*Microcystis* sp.11	UDL	UDL	UDL	UDL	UDL	UDL	UDL	UDL	UDL	UDL
12	*Microcystis* sp.12	UDL	UDL	UDL	UDL	UDL	UDL	UDL	UDL	UDL	UDL
13	*Microcystis* sp.13	18.41	123.66	UDL	2.057	UDL	UDL	UDL	UDL	UDL	144.13
14	*Anabaenopsis* sp.1	UDL	UDL	UDL	UDL	UDL	UDL	UDL	UDL	UDL	UDL
15	*Anabaenopsis* sp.2	UDL	UDL	UDL	UDL	UDL	UDL	UDL	UDL	UDL	UDL
16	*Anabaenopsis* sp.3	UDL	UDL	UDL	UDL	UDL	UDL	UDL	UDL	UDL	UDL
17	*Anabaenopsis* sp.4	UDL	UDL	UDL	UDL	UDL	UDL	UDL	UDL	UDL	UDL
18	*Anabaenopsis* sp.5	UDL	UDL	UDL	UDL	UDL	UDL	UDL	UDL	UDL	UDL
19	*Anabaenopsis* sp.6	UDL	UDL	UDL	UDL	UDL	UDL	UDL	UDL	UDL	UDL
20	*Anabaena* sp. 1	UDL	UDL	UDL	UDL	UDL	UDL	UDL	UDL	UDL	UDL
21	*Anabaena* sp. 2	UDL	UDL	UDL	UDL	UDL	UDL	UDL	UDL	UDL	UDL
22	*Anabaena* sp. 3	UDL	UDL	UDL	UDL	UDL	UDL	UDL	UDL	UDL	UDL
23	*Anabaena* sp. 4	UDL	UDL	UDL	UDL	UDL	UDL	UDL	UDL	UDL	UDL
24	*Planktothrix* sp.1	UDL	UDL	UDL	UDL	UDL	UDL	UDL	UDL	UDL	UDL

UDL: under detection level.

**Table A2:** Microcystin concentration (µg/g DW) in isolated cyanobacteria.

No.	Cyanobacteria	dmRR	RR	NOD	YR	dmLR	LR	LY	LW	LF	Total
1	*Microcystis* sp.1	UDL	UDL	UDL	0	65.68	6773.68	UDL	UDL	UDL	6839.35
2	*Microcystis* sp.2	UDL	UDL	UDL	33.65	30.26	257.98	UDL	UDL	UDL	321.90
3	*Microcystis* sp.3	UDL	UDL	UDL	0	23.03	4418.81	UDL	UDL	UDL	4441.84
4	*Microcystis* sp.4	UDL	UDL	UDL	1377.87	12.74	901.75	UDL	UDL	UDL	2292.36
5	*Microcystis* sp.5	UDL	UDL	UDL	UDL	21.27	9203.45	UDL	UDL	UDL	9224.72
6	*Microcystis* sp.6	UDL	UDL	UDL	UDL	UDL	UDL	UDL	UDL	UDL	UDL
7	*Microcystis* sp.7	UDL	UDL	UDL	UDL	UDL	4.66	UDL	UDL	UDL	4.66
8	*Microcystis* sp.8	UDL	UDL	UDL	UDL	UDL	2.04	UDL	UDL	UDL	2.036
9	*Microcystis* sp.9	87.30	307.47	0.32	6.65	38.95	155.16	UDL	UDL	UDL	595.84
10	*Microcystis* sp.10	UDL	UDL	UDL	UDL	UDL	UDL	UDL	UDL	UDL	UDL
11	*Microcystis* sp.11	UDL	UDL	UDL	UDL	UDL	UDL	UDL	UDL	UDL	UDL
12	*Microcystis* sp.12	UDL	UDL	UDL	UDL	UDL	UDL	UDL	UDL	UDL	UDL
13	*Microcystis* sp.13	182.73	751.11	0.40	22.82	UDL	UDL	1.02	1.40	UDL	959.48
14	*Anabaenopsis* sp.1	UDL	UDL	UDL	UDL	UDL	UDL	UDL	UDL	UDL	UDL
15	*Anabaenopsis* sp.2	UDL	UDL	UDL	UDL	UDL	UDL	UDL	UDL	UDL	UDL
16	*Anabaenopsis* sp.3	UDL	UDL	UDL	UDL	UDL	UDL	UDL	UDL	UDL	UDL
17	*Anabaenopsis* sp.4	UDL	UDL	UDL	UDL	UDL	UDL	UDL	UDL	UDL	UDL
18	*Anabaenopsis* sp.5	UDL	UDL	UDL	UDL	UDL	UDL	UDL	UDL	UDL	UDL
19	*Anabaenopsis* sp.6	UDL	UDL	UDL	UDL	UDL	UDL	UDL	UDL	UDL	UDL
20	*Anabaena* sp. 1	UDL	UDL	UDL	UDL	UDL	UDL	UDL	UDL	UDL	UDL
21	*Anabaena* sp. 2	UDL	UDL	UDL	UDL	UDL	UDL	UDL	UDL	UDL	UDL
22	*Anabaena* sp. 3	UDL	UDL	UDL	UDL	UDL	UDL	UDL	UDL	UDL	UDL
23	*Anabaena* sp. 4	UDL	UDL	UDL	UDL	UDL	UDL	UDL	UDL	UDL	UDL
24	*Planktothrix* sp.1	UDL	UDL	UDL	UDL	UDL	UDL	UDL	UDL	UDL	UDL

UDL: under detection level.

## Appendix C

**Table A3:** Microcystin concentration (µg/g DW) in animals.

No.	Animals	Organ	dmRR	MCRR	NOD	MCYR	dmLR	MCLR	MCLY	MCLW	MCLF	Total
	Fish											
1	Ganges river sprat (*Corica soborna*)	Visceral	UDL	1.29	UDL	UDL	UDL	1.09	UDL	UDL	UDL	2.38
2	Tilapia (*Oreochromis* sp.)	Visceral	UDL	2.87	UDL	UDL	UDL	n.q.	UDL	UDL	UDL	2.87
3	Tilapia (*Oreochromis* sp.)	Visceral	UDL	UDL	UDL	UDL	UDL	0.76	UDL	UDL	UDL	0.76
4	Tilapia (*Oreochromis* sp.)	Gut	UDL	9.91	UDL	UDL	0.36	1.29	UDL	UDL	UDL	11.55
5	Tilapia (*Oreochromis* sp.)	Digestive tract	UDL	UDL	UDL	4.90	1.22	109.83	UDL	UDL	UDL	115.95
6	Tilapia (*Oreochromis* sp.)	Liver	UDL	UDL	UDL	0.27	UDL	13.12	UDL	UDL	UDL	13.39
7	Sknakskin gourami (*Trichogaster pectoralis*)	Visceral	UDL	UDL	UDL	0.02	UDL	13.84	UDL	UDL	UDL	13.86
8	Gold fish (*Carassius* sp.)	Visceral	UDL	UDL	UDL	UDL	UDL	18.57	UDL	UDL	UDL	18.57
9	Gold fish (*Carassius* sp.)	Digestive tract	UDL	UDL	UDL	UDL	UDL	0.36	UDL	UDL	UDL	0.36
10	Suckermouth catfish (*Hypostomus plecostomus*)	Digestive tract	UDL	UDL	UDL	9.08	1.15	98.15	UDL	UDL	UDL	108.38
11	Suckermouth catfish (*Hypostomus plecostomus*)	Liver	UDL	UDL	UDL	0.99	UDL	17.99	UDL	UDL	UDL	18.97
12	Suckermouth catfish (*Hypostomus plecostomus*)	Musscle	UDL	UDL	UDL	0.07	UDL	3.66	UDL	UDL	UDL	3.73
	**Mollusca**											
13	Apple snail (*Pomacea canaliculata*)	Visceral	UDL	UDL	UDL	UDL	0.21	UDL	UDL	UDL	UDL	0.21
14	Apple snail (*Pomacea canaliculata*)	Visceral	UDL	UDL	UDL	UDL	0.33	UDL	UDL	UDL	UDL	0.33
15	Apple snail (*Pomacea canaliculata*)	Visceral	UDL	1.67	UDL	UDL	0.22	UDL	UDL	UDL	UDL	1.89
16	Apple snail (*Pomacea canaliculata*)	Visceral	UDL	UDL	UDL	UDL	0.24	UDL	UDL	UDL	UDL	0.24
17	Apple snail (*Pomacea canaliculata*)	Visceral	UDL	UDL	UDL	UDL	0.36	UDL	UDL	UDL	UDL	0.36
18	Apple snail (*Pomacea canaliculata*)	Visceral	UDL	2.35	UDL	UDL	0.36	UDL	UDL	UDL	UDL	2.71
19	Apple snail (*Pomacea canaliculata*)	Visceral	UDL	2.61	UDL	UDL	0.37	UDL	UDL	UDL	UDL	2.98
20	Apple snail (*Pomacea canaliculata*)	Visceral	UDL	UDL	UDL	UDL	0.25	UDL	UDL	UDL	UDL	0.25
21	Apple snail (*Pomacea canaliculata*)	Visceral	UDL	UDL	UDL	UDL	0.26	UDL	UDL	UDL	UDL	0.26
22	Apple snail (*Pomacea canaliculata*)	Visceral	UDL	UDL	UDL	UDL	0.27	UDL	UDL	UDL	UDL	0.27
23	Apple snail (*Pomacea canaliculata*)	Visceral	UDL	2.75	UDL	UDL	0.46	UDL	UDL	UDL	UDL	3.21
24	Apple snail (*Pomacea canaliculata*)	Visceral	UDL	2.56	UDL	UDL	0.43	UDL	UDL	UDL	UDL	2.99
25	Apple snail (*Pomacea canaliculata*)	Visceral	UDL	UDL	UDL	UDL	0.33	UDL	UDL	UDL	UDL	0.33
26	Apple snail (*Pomacea canaliculata*)	Visceral	UDL	UDL	UDL	UDL	0.28	UDL	UDL	UDL	UDL	0.28
27	Apple snail (*Pomacea canaliculata*)	Visceral	UDL	UDL	UDL	UDL	0.28	UDL	UDL	UDL	UDL	0.28
28	Apple snail (*Pomacea canaliculata*)	Visceral	UDL	UDL	UDL	UDL	UDL	2.56	UDL	UDL	UDL	2.56
29	Apple snail (*Pomacea canaliculata*)	Visceral	UDL	UDL	UDL	0.05	UDL	2.86	UDL	UDL	UDL	2.90
30	Apple snail (*Pomacea canaliculata*)	Visceral	UDL	UDL	UDL	UDL	UDL	1.61	UDL	UDL	UDL	1.61
31	Apple snail (*Pomacea canaliculata*)	Egg	UDL	UDL	UDL	UDL	0.23	UDL	UDL	UDL	UDL	0.23
32	Apple snail (*Pomacea canaliculata*)	Musscle	UDL	UDL	UDL	UDL	0.15	UDL	UDL	UDL	UDL	0.15
33	Apple snail (*Pomacea canaliculata*)	Musscle	UDL	UDL	UDL	UDL	0.23	UDL	UDL	UDL	UDL	0.23
34	Apple snail (*Pomacea canaliculata*)	Musscle	UDL	UDL	UDL	UDL	0.30	UDL	UDL	UDL	UDL	0.30
35	Apple snail (*Pomacea canaliculata*)	Whole neonates	UDL	UDL	UDL	UDL	0.25	UDL	UDL	UDL	UDL	0.25
	**Crustaceae**								UDL	UDL	UDL	
36	White leg shrimp (*Litopenaeus vannamei*)	Whole head	UDL	14.43	UDL	UDL	UDL	0.79	UDL	UDL	UDL	15.21

UDL: under detection level.
